# Precision of the transpulmonary thermodilution measurements

**DOI:** 10.1186/cc10421

**Published:** 2011-08-27

**Authors:** Xavier Monnet, Romain Persichini, Mariem Ktari, Mathieu Jozwiak, Christian Richard, Jean-Louis Teboul

**Affiliations:** 1 AP-HP, Hôpitaux Universitaires Paris-Sud, service de réanimation médicale, Le Kremlin-Bicêtre, F-94270 France; 2 Univ Paris-Sud, Faculté de médecine Paris-Sud, EA 4046, Le Kremlin-Bicêtre, F-94270 France

## Abstract

**Introduction:**

We wanted to determine the number of cold bolus injections that are necessary for achieving an acceptable level of precision for measuring cardiac index (CI), indexed global end-diastolic volume (GEDVi) and indexed extravascular lung water (EVLWi) by transpulmonary thermodilution.

**Methods:**

We included 91 hemodynamically stable patients (age 59 (25% to 75% interquartile range: 39 to 79) years, simplified acute physiologic score (SAPS)II 59 (53 to 65), 56% under norepinephrine) who were monitored by a PiCCO2 device. We performed five successive cold saline (15 mL, 6°C) injections and recorded the measurements of CI, GEDVi and EVLWi.

**Results:**

Considering five boluses, the coefficient of variation (CV, calculated as standard deviation divided by the mean of the five measurements) was 7 (5 to 11)%, 7 (5 to 12)% and 7 (6 to 12)% for CI, GEDVi and EVLWi, respectively. If the results of two bolus injections were averaged, the precision (2 × CV/√ number of boluses) was 10 (7 to 15)%, 10 (7 to 17)% and 8 (7 to 14)% for CI, GEDVi and EVLWi, respectively. If the results of three bolus injections were averaged, the precision dropped below 10%, that is, the cut-off that is generally considered as acceptable (8 (6 to 12)%, 8 (6 to 14)% and 8 (7 to 14)% for CI, GEDVi and EVLWi, respectively). If two injections were performed, the least significant change, that is, the minimal change in value that could be trusted to be significant, was 14 (10 to 21)%, 14 (10 to 24)% and 14 (11 to 23)% for CI, GEDVi and EVLWi, respectively. If three injections were performed, the least significant change was 12 (8 to 17)%, 12 (8 to 19)% and 12 (9 to 19)% for CI, GEDVi and EVLWi, respectively, that is, below the 15% cut-off that is usually considered as clinically relevant.

**Conclusions:**

These results support the injection of at least three cold boluses for obtaining an acceptable precision when transpulmonary thermodilution is used for measuring CI, GEDVi and EVLWi.

## Introduction

Transpulmonary thermodilution (TPTD) is increasingly used in the clinical area [1], but its precision for measuring CI and the number of cold boluses that must be replicated is a matter of debate. Indeed, a recent study concluded that calculating the mean of two TPTD measurements was enough for reaching an acceptable level of precision [2] but it included a limited number of patients. Additionally, for another transpulmonary dilution technique using the lithium and not the cold dilution, it has been recently shown that at least three dilution measurements were required for reaching an acceptable precision. In addition to CI, TPTD also allows estimating the global end-diastolic volume (GEDV, that is, the volume of the cardiac cavities at end-diastole) and the extravascular lung water (EVLW, that is, the volume of the pulmonary edema) [3]. The precision of EVLW measurement derived from TPTD has been reported by some studies [2,4-6], but again, the number of cold injectates that is needed for obtaining an acceptable reproducibility of the measurements remains to be determined. As for GEDV, its precision has been investigated in one study only [2].

Thus, we attempted to answer the important practical question to know the number of thermal injections that must be performed for assessing CI, GEDV and EVLW with an acceptable precision. In particular, we evaluated the number of thermodilution measurements that must be replicated for detecting changes in CI, GEDV and EVLW ≥15% with an acceptable confidence. We also analyzed the factors influencing the precision of the measurements.

## Materials and methods

### Patients

This prospective study was conducted in the 15-bed intensive care unit of a university hospital. As approved by the Institutional Review Board of our institution, patients were included according to an emergency procedure. A deferred informed consent was asked from the patient's surrogate as soon as possible. As he/she recovered consciousness, a deferred informed consent was asked from the patient. If the patient or his/her next of kin refused to consent, the patient's data were not entered into analysis.

Patients were included if they had a femoral arterial catheter (Pulsiocath PV2015L20N, Pulsion Medical Systems, Munich, Germany) and an internal jugular catheter in place and were routinely monitored by a PiCCO2 device (Pulsion Medical Systems, Munich, Germany). Patients were excluded if they were less than 18 years old and if haemodynamic instability did not allow the mean arterial pressure to remain stable (changes by more than 10%) during at least five minutes before starting the study. Patients with cardiac arrhythmias were not excluded. No patient had a pacemaker.

### Study design and measurements

Immediately after inclusion, one of the investigators (RP) injected five successive cold boluses, each according to the manufacturer's recommendation [7]. For each bolus, we injected 15 mL 0.9% saline at 6°C through the distal port of the internal jugular catheter. The injection was performed as rapidly as possible, irrespective of the respiratory cycle. The injectate temperature was carefully checked to be <6°C for all boluses, as displayed by the PiCCO device. For ensuring that boluses were <6°C, we used two packs of saline, one frozen and one at 6°C. For each bolus, we sampled 20 mL from the 6°C saline pack, injected it into the iced pack and re-sampled 15 mL from this saline that had been cooled by the contact with ice. These 15 mL were used for performing the bolus. The thermodilution curve recorded by the arterial thermistance was automatically analyzed by the PiCCO2 device, allowing obtaining the value of cardiac output, of GEDV indexed for body surface (GEDVi) and of EVLW indexed for predicted body weight (EVLWi). The five boluses were performed one after another, as soon as blood temperature had returned to its baseline value, as indicated by the device. The values of CI, GEDVi and EVLWi obtained from each thermodilution were collected. No thermodilution curve was rejected from analysis. Treatments were kept unchanged and patients were not mobilized during the study period. All measurements were performed by the same operator (RP).

### Data analysis

In each patient, we calculated the coefficient of variation (CV) of the TPTD variables (CI, GEDVi and EVLWi). The CV is a normalized measure of dispersion of a probability distribution. It was calculated as being the standard deviation divided by the mean of the five measurements. This relatively large number of measurements thus allowed obtaining a reliable value of CV. The coefficient of error (CE) was obtained by using the formula CE = CV/√n, were n was the number of replicates of measurements in each patient. The precision was calculated as being two CV for a single measurement and two CE for averaged measurements. It is usually considered that a measurement precision level ≤10% is desirable [8]. The least significant change (LSC) is the minimum change that needs to be measured by a device in order to recognize a real change of measurement [9]. The LSC was calculated using the following equation: LSC = CE × 1.96 × √2.

All data except CE, precision, LSC and the dose of norepinephrine were normally distributed (Kolmogorov-Smirnov test for normality) and were expressed as median (25 to 75% interquartile range). As we initially planned to divide our population into five different subgroups depending upon the value of CI, we planned to include a sufficient number of patients (100) for reaching a normal distribution of CI in all subgroups. A *P-*value <0.05 was considered statistically significant. The statistical analysis was performed with MedCalc 8.1.0.0 (Mariakerke, Belgium).

## Results

### Patients' characteristics

A hundred different patients were initially included in the study. Their characteristics are detailed in Table 1. In nine patients, the TPTD could not measure any variable, likely due to low CI, as suggested by the values observed during the preceding hours. The PiCCO device was instituted due to septic shock in 80% of patients in all patients and it was in place since 28 (5 to 50) hours. Atrial fibrillation was observed in 20% of patients (Table 1). Eighty-three percent of patients were under mechanical ventilation and 74% were sedated. A spontaneous breathing activity was observed in 23 (26%) patients. Eight patients were under continuous veno-venous hemofiltration for renal replacement therapy. The dialysis catheter was inserted in the jugular vein and the blood pump flow was 300 mL/minute.

**Table 1:** Patients characteristics at baseline

Age (years, median (25% to 75% IQR))	59 (39 to 79)
Gender (M/F)	65/26
SAPS II (median (25% to 75% IQR))	59 (53 to 65)
Height (cm, median (25% to 75% IQR))	175 (170 to 180)
Weight (kg, median (25% to 75% IQR))	80 (68 to 92)
Cause of shock	
septic (n, %)	73 (80)
cardiogenic (n, %)	10 (11)
hypovolemic (n, %)	8 (9)
Catecholamines	
norepinephrine (n, %)	51 (56)
dobutamine (n, %)	7 (8)
Heart rate (beats/minute, median (25% to 75% IQR))	86 (70 to 99)
Mean arterial pressure (mmHg, median (25% to 75% IQR))	80 (72 to 90)
Body temperature (°C, median (25% to 75% IQR))	37.1 (36.4 to 37.5)
Cardiac rhythm	
sinus rhythm (n, %)	72 (79)
atrial fibrillation (n, %)	18 (20)
frequent atrial extrasystoles (n, %)	1 (1)
Cardiac index (L/minute/m^2^, median (25% to 75% IQR))	3.5 (2.7 to 4.3)
EVLWi (mL/kg, median (25% to 75% IQR))	9 (8 to 14)
GEDVi (mL/m^2^, median (25% to 75% IQR))	812 (705 to 932)

N = 91

Data are expressed as median (25% to 75% interquartile range (IQR)) or as n (%).

SAPS, Simplified Acute Physiologic Score; EVLWi, extravascular lung water indexed for predicted body weight; GEDVi, global end-diastolic volume indexed for body surface.

### Precision of the TPTD measurements

**Figure 1:**
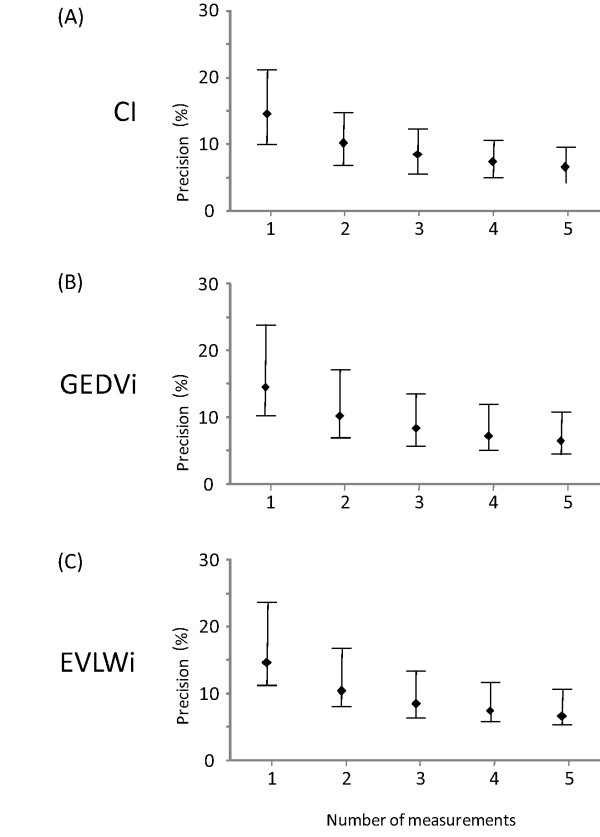
**Relationship between the number of measurements and the precision of transpulmonary thermodilution variables**. CI: cardiac index, GEDVi: global end-diastolic volume indexed for body surface, EVLWi: extravascular lung water indexed for predicted body weight. For instance, if a precision of ±10% would be desired, one would have to take the mean of three measurements. (Data are expressed as medians and 25 to 75% interquartile ranges).

Considering five boluses, the average CI was 3.5 (2.7 to 4.3) L/minute/m^2^, the GEDVi was 812 (708 to 932) mL/m^2 ^and the EVLWi was 9 (8 to 14) mL/kg (Table 1). The precision for one single measurement was 14 (10 to 21)% for CI, 15 (10 to 24)% for GEDVi and 15 (11 to 24)% for EVLWi and the precision for CI was ≥10% in 66 patients. If two boluses were used for TPTD, the precision was reduced to 10 (7 to 15)% for CI, 10 (7 to 17)% for GEDVi, 10 (8 to 17)% for EVLWi (Figure 1) and the precision for CI was ≥10% in 47 patients. If three boluses were used for TPTD, the precision was reduced to 8 (6 to 12)% for CI, 8 (6 to 14)% for GEDVi, 8 (7 to 14)% for EVLWi (Figure 1) and the precision for CI was ≥10% in 33 patients.

**Figure 2:**
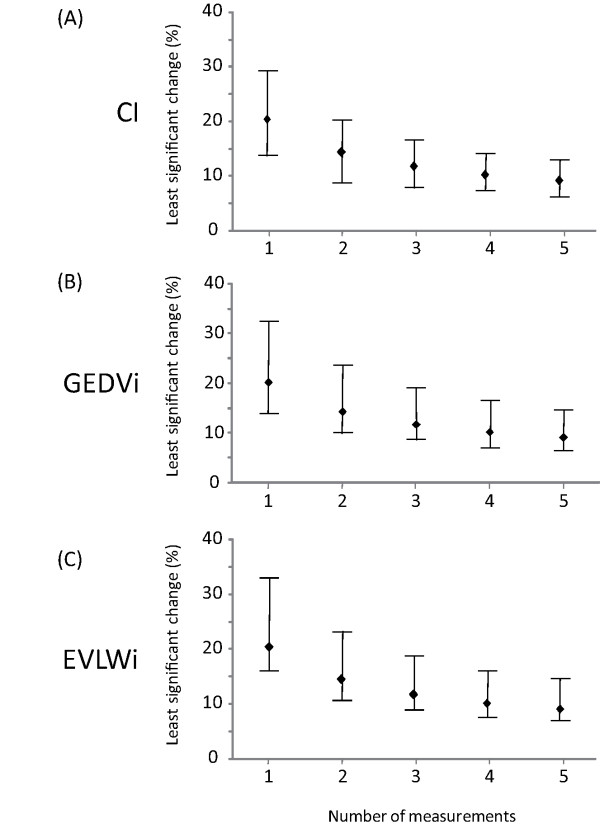
**Relationship between the number of measurements and the least significant change of transpulmonary thermodilution variables**. CI: cardiac index, GEDVi: global end-diastolic volume indexed for body surface, EVLWi: extravascular lung water indexed for predicted body weight. For instance, for assessing a 15% change in CI, GEDVi or EVLWi with a 95% confidence, one would have to take the mean of three measurements. (Data are expressed as medians and 25 to 75% interquartile ranges).

The LSC (for one single measurement) was 20 (14 to 29)% for CI, 20 (14 to 34)% for GEDVi and 20 (16 to 33)% for EVLWi. If two boluses were used for TPTD, the LSC was reduced to 14 (10 to 21)% for CI, 14 (10 to 24)% for GEDVi and 14 (11 to 23)% for EVLWi (Figure 2). If three boluses were used for TPTD, the LSC was 12 (8 to 17)% for CI, 12 (8 to 19)% for GEDVi and 12 (9 to 19)% for EVLWi (Figure 2).

### Factors influencing the precision of the transpulmonary measurements

Excluding the eight patients under continuous veno-venous hemofiltration did not significantly change CV, neither for CI (8 (6 to 12) vs. (8 (6 to 12) for three measurements) nor for GEDVi (8 (5 to 13) vs. 8 (5 to 13) for three measurements) and EVLWi (8 (6 to 14) vs. 8 (6 to 14) for three measurements). Excluding the 23 patients with spontaneous breathing activity did not significantly change CV, neither for CI (8 (5 to 12) vs. (8 (6 to 12) for three measurements) nor for GEDVi (8 (5 to 13) vs. 8 (5 to 13) for three measurements) and EVLWi (8 (6 to 13) vs. 8 (6 to 14) for three measurements). There was no significant correlation between the dose of norepinephrine and the precision of CItd (*P *= 0.06), GEDVi (*P *= 0.10) and EVLWi (*P *= 0.91).

## Discussion

This study shows that three thermodilution measurements are required for estimating CI, GEDVi and EVLWi through TPTD with an acceptable level of precision. If three thermodilution measurements are averaged, the technique allows detecting changes in CI, GEDVi and EVLWi of more than 15% with an acceptable confidence.

There have been a number of studies reporting a good agreement between the measurement of CI by TPTD and by bolus pulmonary artery thermodilution [10-16] or by the Fick method [17,18]. By showing bias and limits of agreement of the TPTD CI compared to the reference CI, these previous studies investigated the technique accuracy [1], that is, its ability to give a value of CI that is close to the reference value [9]. Nevertheless, validation of a technique monitoring CI should not only be based upon accuracy. Another key criterion to consider is precision, that is, the ability of the technique to provide values of CI that are close to each other [8]. Indeed, precision is a very important criterion for at least two reasons. First, it determines the least change that can be trusted with confidence as being significant in clinical practice. This is especially important for techniques measuring CI [19], since one is much more interested in variations of CI values over time than to a given CI value. Second, since precision increases with the number of measurements, it conditions the minimal number of measurements that must be replicated in clinical practice.

Our result concerning the precision of the TPTD measurement of CI is in accordance with two previous studies [20,21] but in discrepancy with another one [2]. In particular, we found that averaging two measurements only might be insufficient for reaching an acceptable precision and that three thermodilution measurements are actually needed. However, the latter study [2] included a much more limited number of patients than our study. Interestingly, the precision of CI measurement we found for TPTD was similar to the precision reported by Cecconi *et al*. for another transpulmonary dilution device, the LiDCO system [22]. As the PiCCO device, this technique uses transpulmonary dilution but it uses lithium rather than cold saline as a diluted indicator. The similarity of precision between the two techniques suggests that their precision is more influenced by the transpulmonary dilution technique itself than the nature of the indicator.

We observed that, provided that three thermodilution measurements were averaged, the precision of the TPTD measurement of CI was within the 10% limit that is usually admitted as being desirable [8]. Comparison with the precision of the bolus pulmonary artery thermodilution is somewhat difficult, since the latter was evaluated by only a few studies. In a 20-year-old study, Stetz *et al*. [23] found that precision of pulmonary artery catheter was similar to that we report with TPTD. More recently, Nilsson *et al*. reported a precision as low as 6% when averaging only three pulmonary artery thermodilutions [24]. If confirmed, these results would suggest that pulmonary artery thermodilution is more precise than TPTD.

The precision was almost similar for CI, GEDVi and EVLWi. This is not surprising since their measurement by TPTD share some common components. We could not analyze some constituents on which the measurement of CI, GEDVi and EVLWi is based, such as the mean transit time and down slope time of the thermodilution curve, what must be considered as a limitation of our study. Importantly, we found that the precision of TPTD measurements was not different when patients with cardiac arrhythmias, continuous veno-venous hemofiltration or spontaneous breathing were excluded from analysis. The absence of significant correlation between the dose of norepinephrine and the precision of CI, GEDVi and EVLWi suggests that vasopressors also do not influence the precision of TPTD measurements.

The good precision of EVLWi and GEDVi measurements is important as an increasing number of studies have proposed to consider EVLW as a prognostic factor [4,5,25] or as a criterion of judgement for therapy [6] in acute respiratory distress syndrome, even though concerns have been raised concerning its reliability in this condition [26]. The results of the present study might have some important implications. The first ones are for daily clinical practice. By showing that injecting three cold boluses is sufficient for obtaining an acceptable precision of the technique, the study supports the manufacturer's guidelines of averaging three thermodilution measurements [7], a recommendation that, until now, was not based upon published data. If one considers that a precision of 8% is sufficient for CI, GEDVi and EVLWi, what is highly reasonable [27], repeating more than three boluses is useless. This might be important when considering that repeating cold injections is time consuming and that thermodilution must be frequently repeated in case of hemodynamic instability [28]. The second implication of the results is for clinical research purposes. For instance, it indicates that if three thermodilution measurements are averaged, a change in CI, GEDVi or EVLWi of 15% or more can be considered as the cut-off defining a positive response to a therapeutic intervention [21,29,30].

We acknowledge some limitations to our study. First, we did not exclude any measurement from analysis, while less skilled operators should do it when obtaining less reliable thermodilution curves. Second, the measurements were performed by only one skilled operator, precluding the assessment of inter-observer variability. Third, we did not investigate whether injecting more than 15 mL for performing each bolus could increase the precision of the technique. Fourth, the body temperature, CI, GEDVi and EVLWi values were within the normal range, such that the precision of TPTD for extreme values of these variables remains to be determined. In addition, we did not include patients with slow atrial fibrillation, which may affect the precision of TPTD measurements. Also, ideally, the measurements should have consisted of a series of five boluses compared to another series of five boluses for which one to five are randomly selected. Finally the precision of TPTD when using a humeral arterial catheter remains to be determined.

## Conclusions

We found that three thermodilution measurements are needed for estimating CI, GEDVi and EVLWi with TPTD. If three thermodilutions measurements were averaged, this allowed detecting a 12% change in CI, GEDVi and EVLWi with a 95% certainty. In addition to the accuracy of the technique, which has been already reported, these results may reinforce the level of evidence that TPTD is a reliable technique for monitoring critically ill patients.

## Key messages

• Three thermodilution measurements are needed for estimating cardiac index, global end-diastolic volume and extravascular lung water with transpulmonary thermodilution

• If three thermodilution measurements are averaged, this allows detecting a 12% change in estimating cardiac index, global end-diastolic volume and extravascular lung water with a 95% certainty

## Abbreviations

CI: cardiac index; EVLWi: extravascular lung water indexed for predicted body weight; GEDVi: global end-diastolic volume indexed for body surface; SAPS: simplified acute physiologic score; TPTD: transpulmonary thermodilution.

## Competing interests

Profs. Jean-Louis Teboul and Xavier Monnet are members of the Medical Advisory Board of Pulsion Medical Systems. As consultants for this company, they received honoraria. The company did not finance the manuscript. The company was not involved in any part of the conception or performance of the study. The other authors declare that they have no conflict of interest.

## Authors' contributions

XM conceived the study, performed analysis and interpretation of the data, and drafted the manuscript. RP performed the collection of data, contributed to analysis and interpretation of the data and helped draft the manuscript. MK performed the collection of data, contributed to analysis and interpretation of the data, and helped draft the manuscript. MJ contributed to the collection of data, CR participated in the design of the study, contributed to analysis and interpretation of the data and helped draft the manuscript. J-LT conceived the study, participated in its design, contributed to analysis and interpretation of the data and helped draft the manuscript. All authors read and approved the final manuscript.
